# Supplementary material for: Neurotherapeutic Effect of Inula britannica var. Chinensis against H2O2-Induced Oxidative Stress and Mitochondrial Dysfunction in Cortical Neurons
Source: Antioxidants (Basel). 2021 Mar 3;10(3):375. doi: 10.3390/antiox10030375 (PMC8001496; doi:10.3390/antiox10030375)
Supplement: Supplementary file 1 [file antioxidants-10-00375-s001.pdf]

Supplementary of antioxidants-1096936

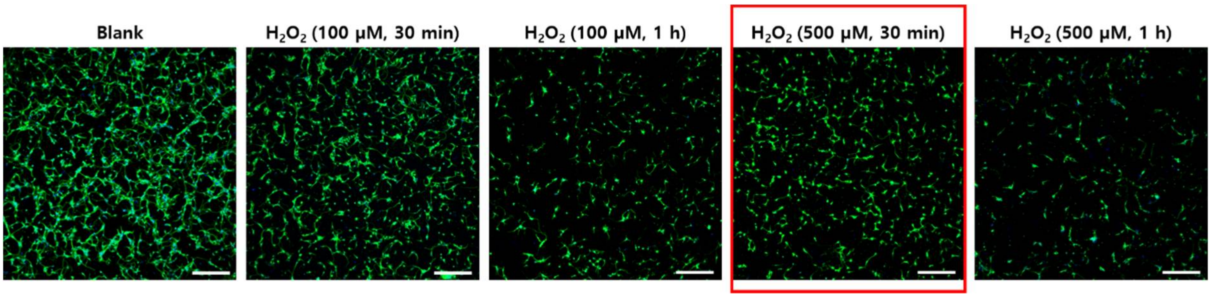

FigureS1. Immunocytochemical analysis of cortical neurons to assess the H_2O_2 optimal concentration and incubation time**.** Representative immunocytochemical images showing Tuj1 (green) in cortical neurons treated with 100 or 500 μM H_2O_2 for 30 min or 1 h. White scale bar = 200 μm.

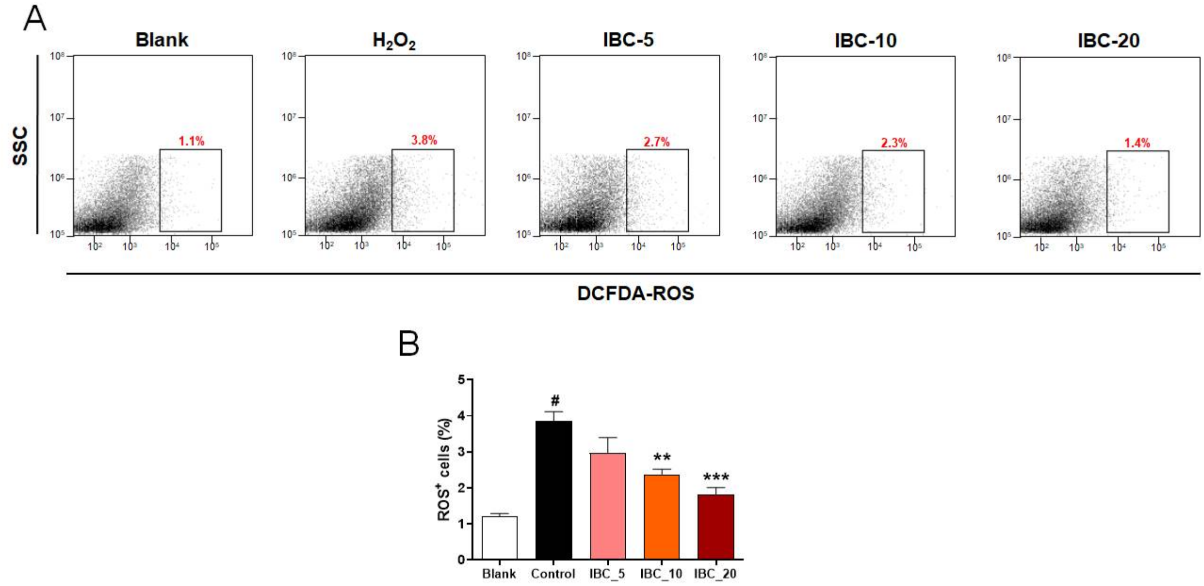

FigureS2. Flow cytometric analysis of intracellular ROS in H_2O_2-treated cortical neuron with 5, 10 and 20 μg/ml of IBC**.** (**A**) Representative flow cytometric dot plots graphs showing DCFDA-ROS expression. (**B**) Quantification of ROS-positive cells at the single cell level. Data represent mean ± SEM of six independent experiments. Significant differences indicated as *# $p < 0.001$* compared vs. the blank group, *** $p < 0.01$ and *** $p < 0.001$* vs. the H_2O_2 group were analyzed via one-way ANOVA with Tukey's post-hoc test.

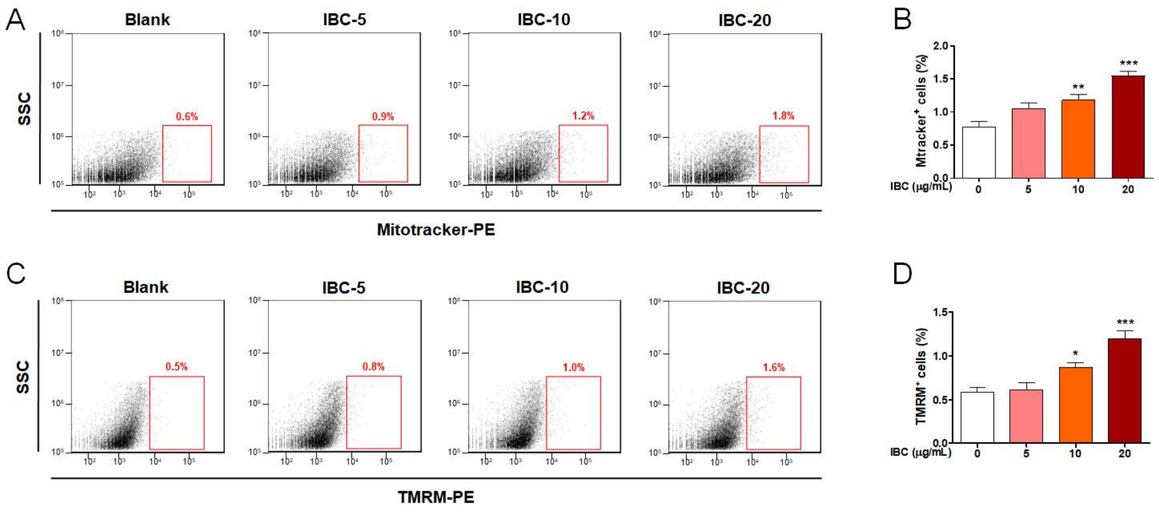

FigureS3. Mitotracker (Mtracker) and TMRM Flow cytometry analysis of cortical neuron treated with 5, 10 and 20 μg/ml of IBC without H_2O_2 exposure. (**A**) Representative dot plots graphs showing Mtracker expression. (**B**) Quantification of Mtracker-positive cells at the single cell level. (**C**) Representative dot plots graphs showing TMRM expression. (**D**) Quantification of TMRM-positive cells at the single cell level. Data represent mean ± SEM of six independent experiments. Statistically significant differences compared with blank group (0 μg/ml) by one-way ANOVA with Tukey's post-hoc test. (*$p < 0.05$; **$p < 0.01$; ***$p < 0.001$*).

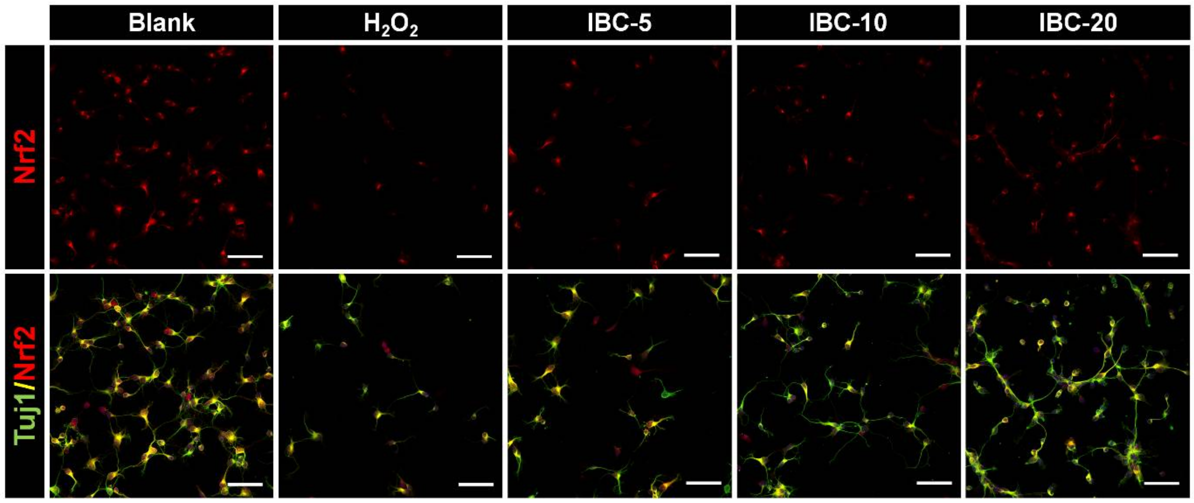

FigureS4. Nrf2 immunocytochemical analysis of cortical neuron treated with 5, 10 and 20 μg/ml of IBC with H_2O_2 exposure. Representative immunocytochemical images showing Nrf2 (red) and Tuj1 (green) in H_2O_2-treated cortical neurons with 5, 10 and 20 μg/ml of IBC. White scale bar = 50 μm.

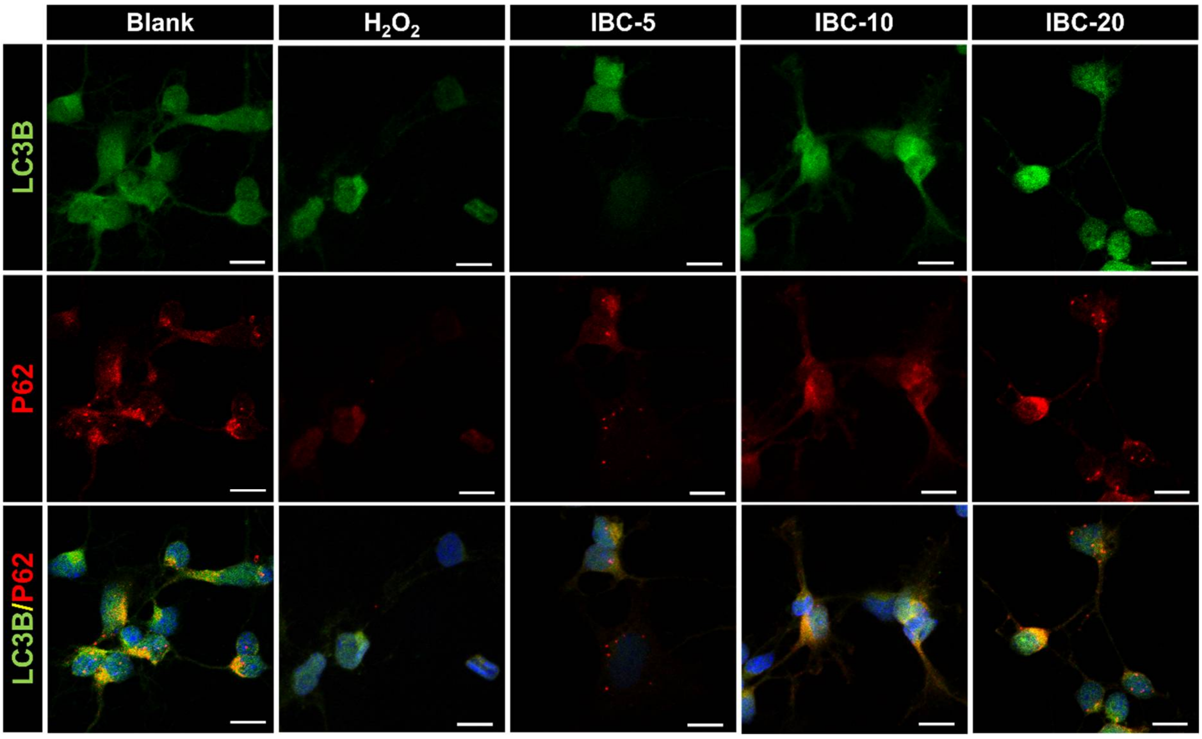

FigureS5. LC3B/P62 immunocytochemical analysis of cortical neurons treated with 5, 10 and 20 μg/ml of IBC with H_2O_2 exposure. Representative immunocytochemical images showing LC3B (green) and p62 (red) in H_2O_2-treated cortical neurons with 5, 10 and 20 μg/ml of IBC. White scale bar = 15 μm.

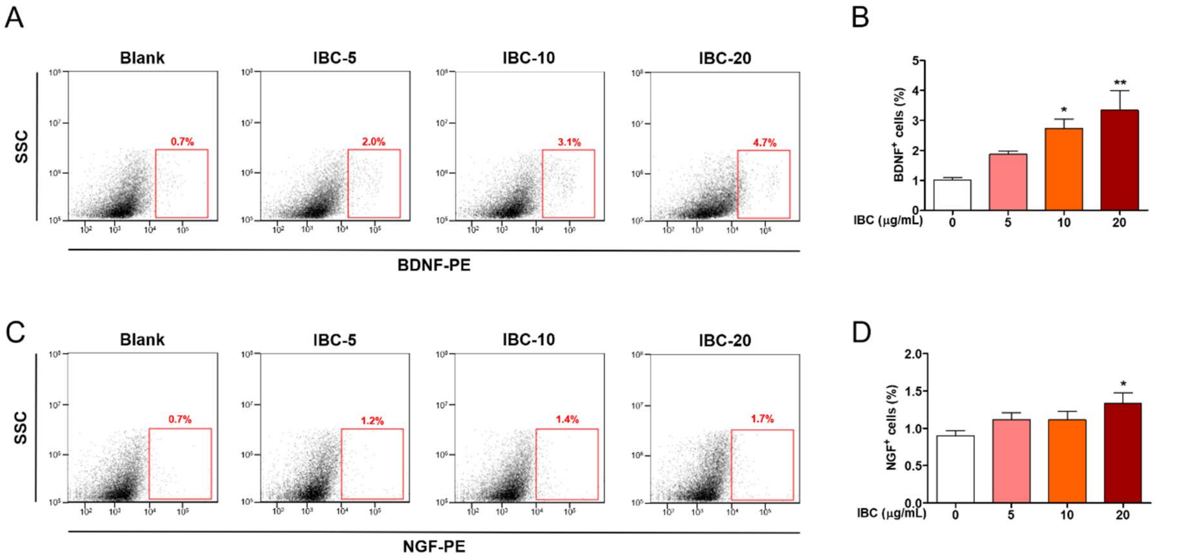

FigureS6. BDNF and NGF Flow cytometry analysis of cortical neuron treated with 5, 10 and 20 μg/ml of IBC without H_2O_2 exposure. (**A**) Representative dot plots graphs showing BDNF expression. (**B**) Quantification of BDNF-positive cells at the single cell level. (**C**) Representative dot plots graphs showing NGF expression. (**D**) Quantification of NGF-positive cells at the single cell level. Data represent mean ± SEM of six independent experiments. Statistically significant differences compared with blank group (0 μg/ml) by one-way ANOVA with Tukey's post-hoc test. (*$*p < 0.05$; $**p < 0.01$*).
